# Suppressive action of nesfatin-1 and nesfatin-1-like peptide on cortisol synthesis in human adrenal cortex cells

**DOI:** 10.1038/s41598-024-54758-7

**Published:** 2024-02-17

**Authors:** Atefeh Nasri, Jade Sands, Suraj Unniappan

**Affiliations:** Laboratory of Integrative Neuroendocrinology, Department of Veterinary Biomedical Sciences, Western College of Veterinary Medicine, University of Saskatchewan, 52 Campus Drive, Saskatoon, SK S7N 5B4 Canada

**Keywords:** Nucleobindin, Stress, Cortisol, Nesfatin-1-like peptide, Nesfatin-1, Mouse, Human, Physiology, Endocrinology

## Abstract

Nucleobindin-derived peptides, nesfatin-1 [NESF-1] and nesfatin-1-like-peptide [NLP] have diverse roles in endocrine and metabolic regulation. While both peptides showed a stimulatory effect on the synthesis of proopiomelanocortin (POMC), the adrenocorticotropic hormone (ACTH) precursor in mouse corticotrophs, whether NESF-1 and NLP have any direct effect on glucocorticoid [GC] synthesis in the adrenal cortex remains unknown. The main aim of this study was to determine if NESF-1 and/or NLP act directly on adrenal cortex cells to regulate cortisol synthesis in vitro. Whether NLP injection affects stress-hormone gene expression in the adrenal gland and pituitary in vivo in mice was also assessed. In addition, cortisol synthetic pathway in *Nucb1* knockout mice was studied. Human adrenal cortical [H295R] cells showed immunoreactivity for both NUCB1/NLP and NUCB2/NESF-1. NLP and NESF-1 decreased the abundance of steroidogenic enzyme mRNAs, and cortisol synthesis and release through the AC/PKA/CREB pathway in H295R cells. Similarly, intraperitoneal injection of NLP in mice decreased the expression of enzymes involved in glucocorticoid (GC) synthesis in the adrenal gland while increasing the expression of *Pomc*, *Pcsk1* and *Crhr1* in the pituitary. Moreover, the melanocortin 2 receptor *(Mc2r)* mRNA level was enhanced in the adrenal gland samples of NLP injected mice. However, the global genetic disruption in *Nucb1* did not affect most steroidogenic enzyme mRNAs, and *Pomc*, *Pcsk2* and *Crhr1* mRNAs in mice adrenal gland and pituitary gland, respectively. Collectively, these data provide the first evidence for a direct inhibition of cortisol synthesis and secretion by NLP and NESF-1*.* NUCB peptides might still elicit a net stimulatory effect on GC synthesis and secretion through their positive effects on ACTH-MC2R pathway in the pituitary.

## Introduction

In recent years, nesfatin-1 [NESF-1; processed from nucleobindin-2/NUCB2] has received much attention due to its roles in metabolism, stress, and anxiety. NESF-1 and nesfatin-1-like-peptide (NLP) affect synthesis of stress hormones^1,2^. The release of glucocorticoids [GC] as end products in the stress/HPA axis modulates metabolism, potentially impacting energy availability through catabolic processes. In animal models, corticosterone was shown to increase the intake of palatable foods, including carbohydrates and lard^3,4^. These findings might explain how repeated stress-related GC secretion leads to the intake of high-calorie food and weight gain. Animals vulnerable to obesity had higher circulating GC, and GC antagonists prevented/reversed the weight gain in these animals^5,6^. Some human studies reported that abdominal obesity might be associated with elevated GCs in response to stress. Food intake was significantly increased immediately after stress, showing that this response was related to the stress reactivity but not the total secreted cortisol^7^. Furthermore, the infusion of corticotropin-releasing hormone [CRH] at physiological doses significantly increased food intake in humans compared to placebo-injected non-obese adults^8^. CRH initiates the HPA axis. Various neurons expressing NUCB2/NESF-1 are colocalized with CRH in hypothalamic paraventricular nucleus^9^. NUCB2/NESF-1 increased the excitability of CRH neurons^10^. NESF-1 was shown to enhance cytoplasmic Ca2+ levels in CRH neurons^11^. Moreover, bilateral adrenalectomy increased NESF-1 mRNA in the rat hypothalamus^12.^ Less than 50% of neurons expressing NESF-1 in PVN and arcuate nucleus contain glucocorticoid receptors^13^. These findings suggests that central NESF-1 could affect CRH neurons in PVN and initiate central and peripheral HPA axis responses. Two reports showed that NESF-1 might apply its anorectic effect through CRH and its receptor [CRHR]-mediated system^14,15^. In both studies, intracerebroventricular [ICV] injection of NESF-1 decreased food intake, in rats during the dark phase^14^, and in neonatal chicks^15^. However, CRHR antagonists abolished the anorectic effect of NESF-1^14,15^.

Very recently, it has been proposed that a NUCB2-related peptide, nucleobindin-1 [NUCB1], possesses a nesfatin-1-like peptide (NLP) sequence, which could be processed by prohormone convertases. NUCB1 and NUCB2 shared 60% sequence homology in the mouse genome^16^. It was reported that the bioactive core of NLP and NESF-1 shared 76% amino acid sequence homology in mouse^17^. The presence of NLP was reported in tissues in which NUCB2/NESF-1 was previously identified, including pancreas, pituitary, gonads, and gut^17,18^. Similarly, NLP was shown to suppress food intake and modulate the expression of appetite-regulatory hormones in goldfish^18^ and rats^19^.

Previous studies from our lab also showed that NESF-1 is a stress-responsive peptide that stimulates stress-related hypothalamus-pituitary-interrenal [HPI; similar to mammalian HPA tissues] axis hormones in goldfish^2^. Moreover, NESF-1 was shown to directly stimulate the synthesis of ACTH precursor in mouse pituitary corticotrophs^1^. NESF-1 is a catabolic hormone with satiety effects and stimulates ACTH precursor, which is the primary pituitary regulator of another catabolic hormone, cortisol. Moreover, NESF-1 binding sites were detected in the adrenal gland of rats^20^. Based on this evidence, especially the positive roles of NESF-1 or NLP on ACTH, we hypothesized that they elicit a similar stimulatory role on cortisol synthesis and secretion. The objective of this study was to assess whether adrenal cortex cells express NUCB1/NLP and NUCB2/NESF-1 and whether they directly act on adrenal cortical cells to modulate the synthesis of cortisol and modulate steroidogenic enzymes involved in this pathway in vitro and in vivo.

## Results

### H295R cells express Nucb1 and Nucb2

**Figure 1:**
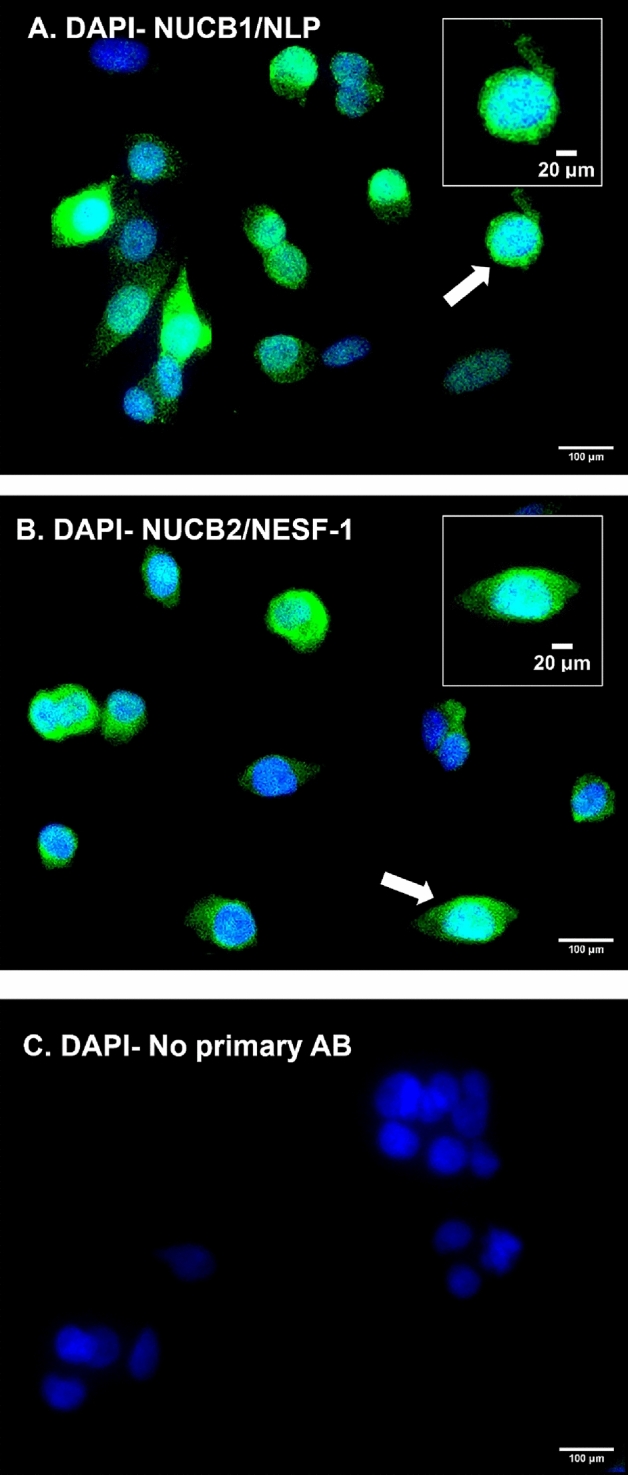
H295R cells are immunoreactive for NUCB1/NLP (**A**) and NUCB2/NESF-1 (**B**), both shown in green color. DAPI stained DNA in blue color group. No primary antibody is negative control group (**C**). Arrows point to magnified cells shown on the top right inset.

Human adrenal cortex (H295R) cells were immunoreactive for both NUCB1/NLP and NUCB2/NESF-1. Both NUCB1/NLP-like and NUCB2/NESF-1-like immunoreactivity showed a diffuse distribution in both cytoplasm [Green] and nucleus [DAPI; blue] in Fig. 1A,B. No immunoreactivity was observed in control groups that were only incubated with the secondary antibodies (Fig. 1C)*.*

### NLP and NESF-1 significantly decreased cortisol content in H295R cells

**Figure 2:**
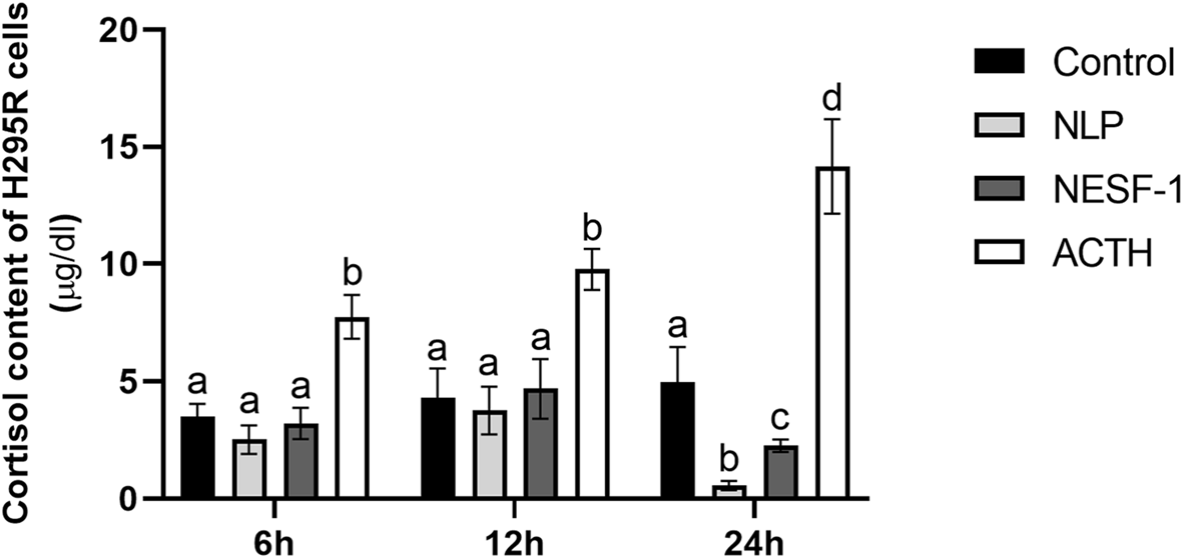
NLP and NESF-1 decreased cellular cortisol content in H295R cells. The results presented are pooled from 3 independent studies with at least triplicates for each treatment. The same alphabets show no significant difference between groups, while different letters indicate significant differences among groups for each time point.

The cortisol content of H295R cells at different time points [6–24 h] is shown in Fig. 2. NLP and NESF-1 at 10 nM decreased cortisol content of H295R cells at 24 h post-incubation, while no such effects were found at other time points tested. Meanwhile, ACTH [100 nM; positive control] enhanced cellular cortisol content at 6, 12 and 24 h post-incubation.

### NLP and NESF-1 decreased cortisol secretion through AC/PKA/CREB-mediated pathway in H295R cells

**Figure 3:**
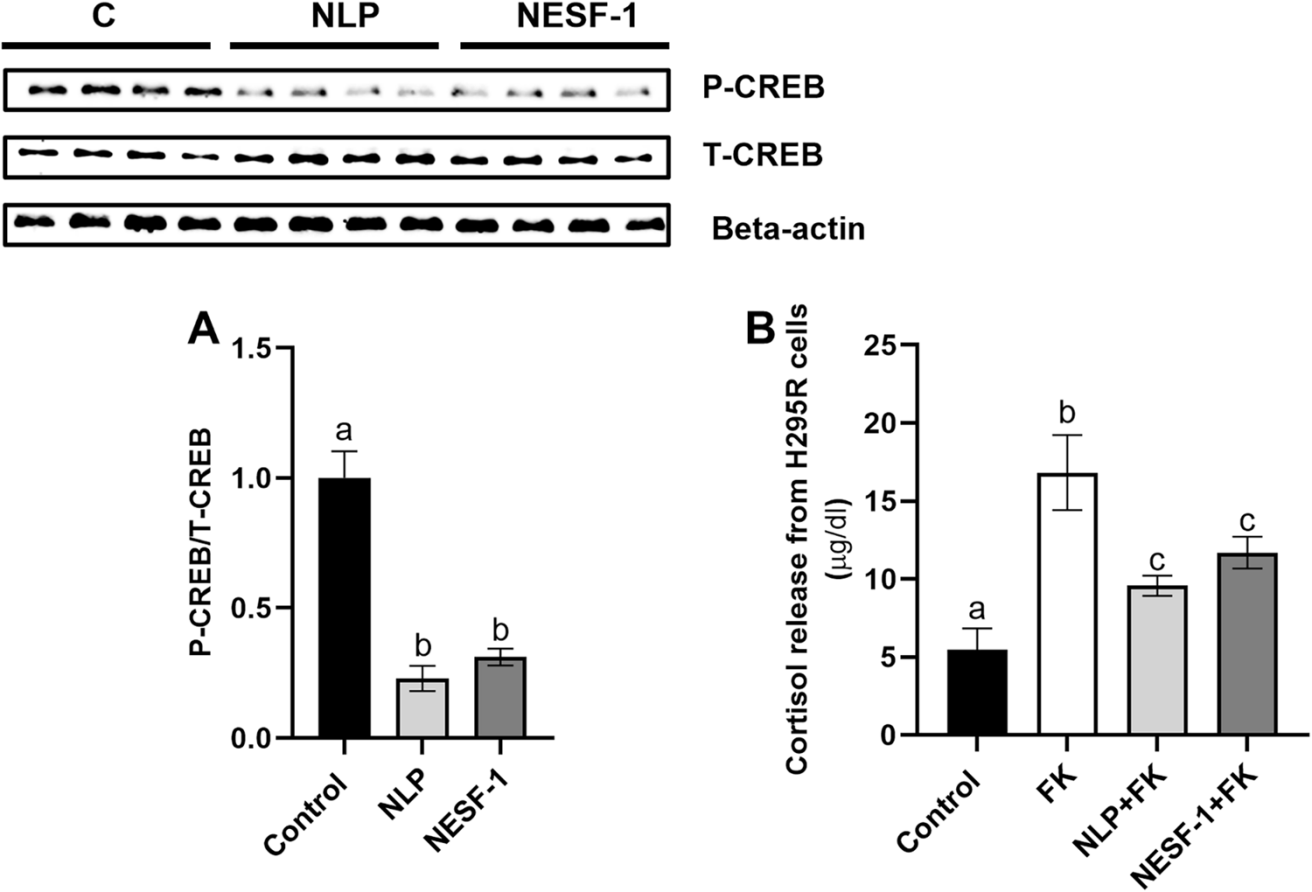
NLP and NESF-1 decreased the phosphorylation of CREB in H295R cells (**A**) and regulated cortisol release through a CREB mediated pathway (**B**). The results presented are pooled from 3 independent studies with duplicate/triplicate for each treatment. Same alphabets show no significant difference between groups, while different letters indicate significant differences among groups. Full size western gel images that served as the source for this figure are shown in Supplementary Fig. 2. Each of the image came from a single source gel image.

As shown in Fig. 3A, NLP and NESF-1 decreased P-CREB/T-CREB ratio by more than twofold compared to the control group. Forskolin enhanced cortisol secretion significantly when compared to the no-treatment control group. When H295R cells were preincubated with either NESF-1 or NLP, the stimulatory effects of forskolin, the classical activator of adenylyl cyclase, on cortisol release was significantly lower compared to the group incubated with forskolin alone (Fig. 3B).

### NESF-1 and NLP decreased the expression of steroidogenic enzymes and endogenous NUCB mRNAs in H295R cells

**Figure 4:**
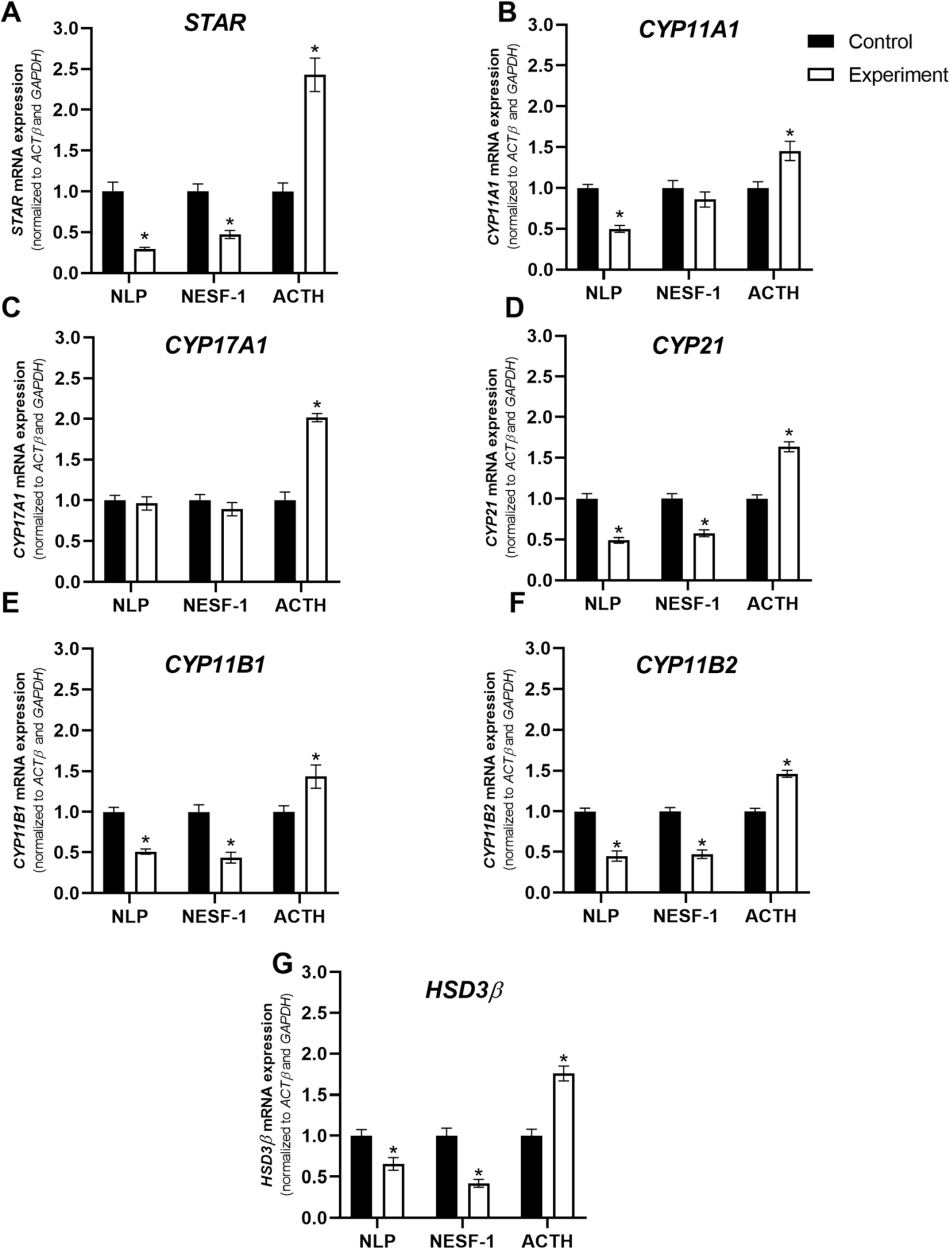
NLP and NESF-1 affected the expression of steroidogenic enzyme mRNA abundance in H295R cells. The results presented are pooled from 3 independent studies with at least triplicates for each treatment. Asterisks show statistical differences between experimental and control groups.

The effects of NESF-1 and NLP on steroidogenic enzyme mRNAs involved in the conversion of cholesterol to biologically active steroid hormones are shown in Fig. 4. NLP and NESF-1 [10 nM] downregulated the abundance of steroidogenic enzyme mRNAs including *STAR*, *CYP21*, *CYP11B1*, *CYP11B2,* and *HSD3Β* at mRNA level at 24 h post-incubation (Fig. 4A and D–G]. *CYP11A1* was downregulated only in the NLP group, while *CYP17A1* was not affected by either peptide (Fig. 4B,C).

**Figure 5:**
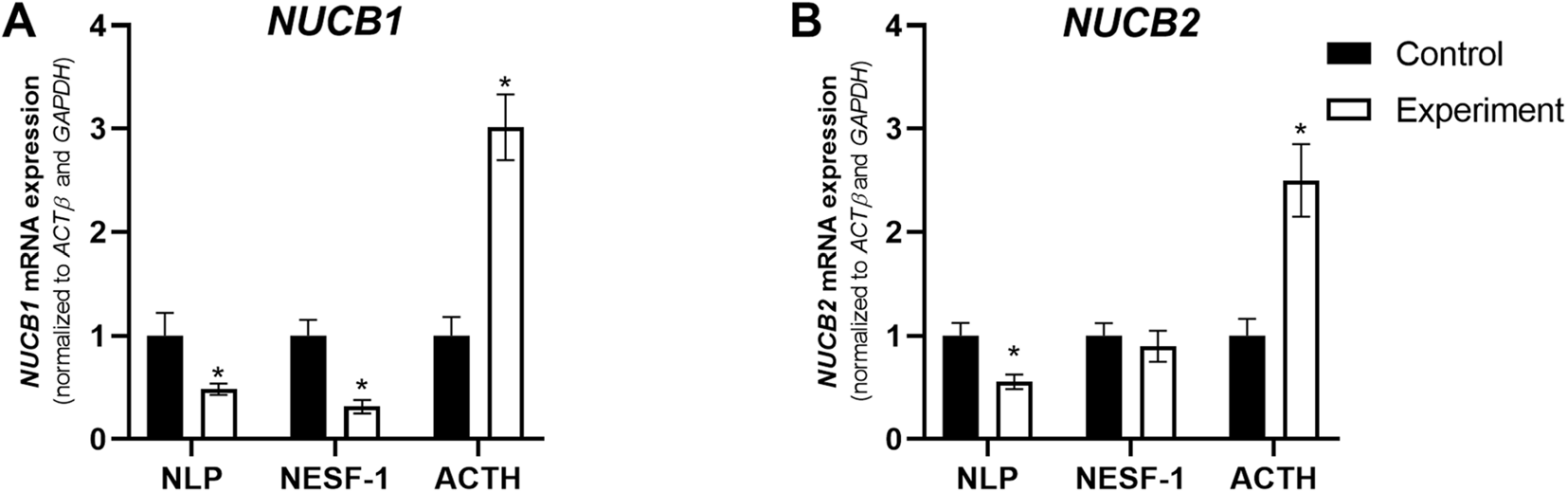
NLP and NESF-1 affected the expression of endogenous *NUCB* mRNAs in H295R cells. The results presented are pooled from 3 independent studies with at least triplicates for each treatment. Asterisks show statistical differences between experimental and control groups.

ACTH [100 nM; positive control] showed the opposite [stimulatory] effect on the expression of all steroidogenic enzymes studied at 24 h post-incubation (Fig. 4A–G). The expression of endogenous *NUCB1* and *NUCB2* mRNAs are shown in Fig. 5A,B. *NUCB1* was downregulated by NLP and NESF-1 while *NUCB2* was downregulated only by NLP. In contrast, ACTH upregulated *NUCB* mRNAs in H295R cells.

### Intraperitoneal [IP] injection of NLP modulated the expression of HPA axis-related enzymes, hormones, receptors and endogenous Nucb at mRNA level in mice

**Figure 6:**
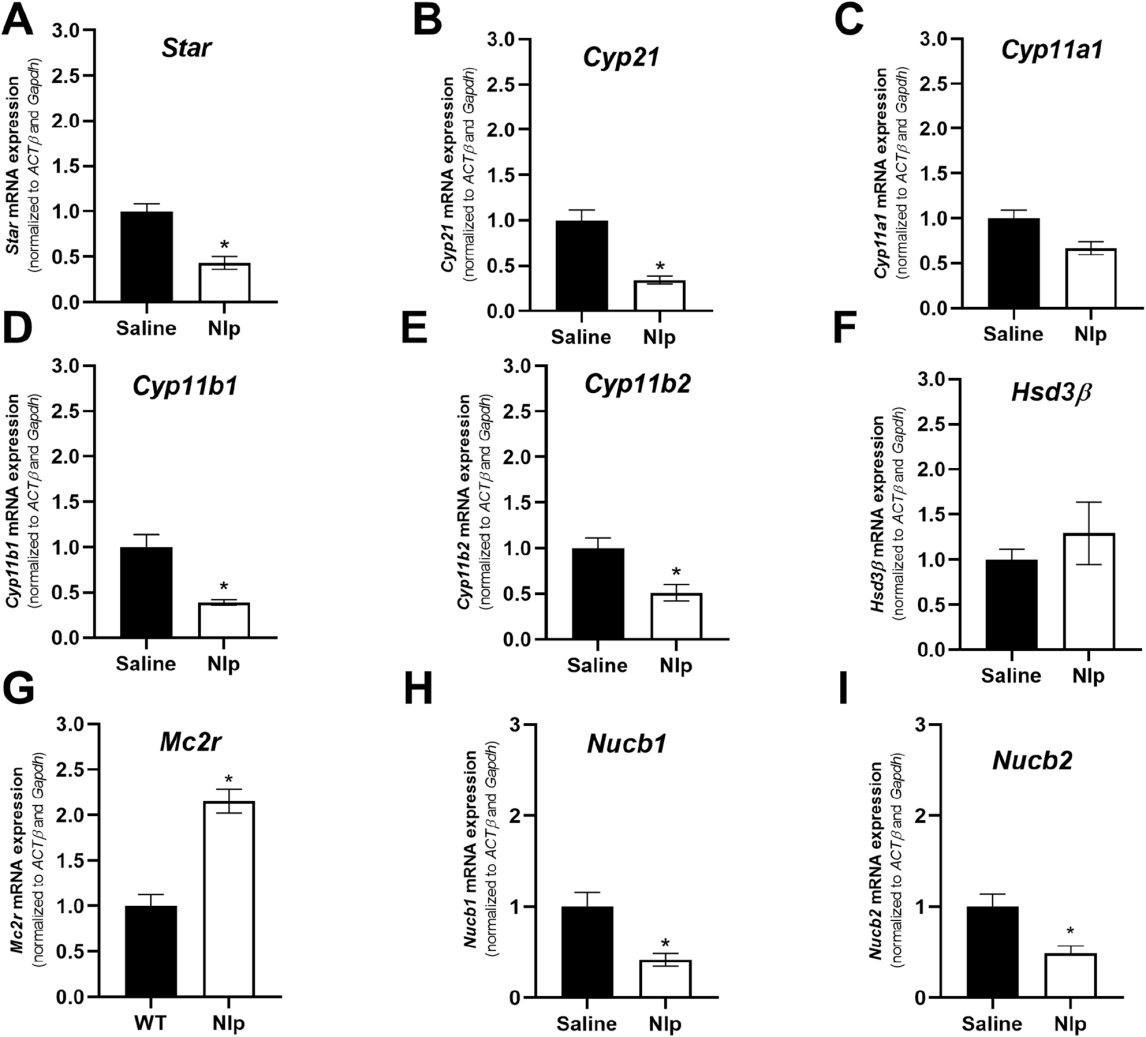
IP injection of 100 µg/Kg BW NLP affected the expression of steroidogenic enzymes, endogenous *Nucb* and *Mc2r* in the mouse adrenal gland. Studies were done on 6–7 tissue samples of mixed-sex C57BL/6 mice. Asterisks show a significant difference between the experimental and control groups.

**Figure 7:**
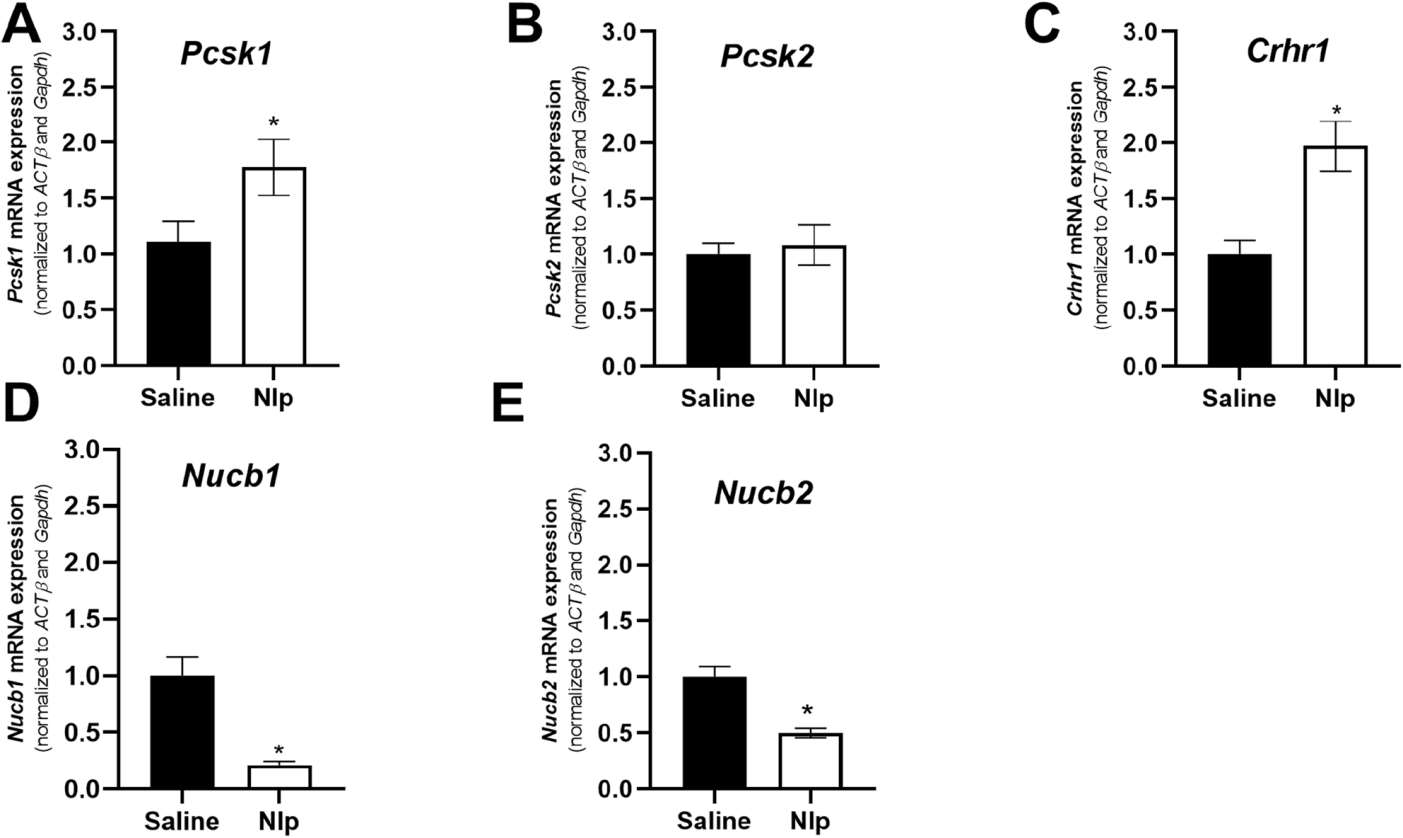
IP injection of 100 µg/Kg BW NLP affected the stress-related genes and endogenous *Nucb* in the mouse pituitary. Studies were done on 6–7 tissue samples of mixed-sex C57BL/6 mice. Asterisks show a significant difference between the experimental and control groups.

IP injection of NLP at 100 µg/kg decreased the expression of steroidogenic enzymes [*Star*, *Cyp21*, *Cyp11b1* and *Cyp11b2*] as well as *Nucb1* and *Nucb2* mRNAs in the adrenal gland (Fig. 6). In contrast, NLP injection enhanced the *Mc2r* mRNA in the adrenal gland of these mice. Furthermore, NLP injection enhanced the expression of *Pomc*, *Pcsk1* and *Crhr1* (Fig. 7A–D) but decreased *Nucb1* and *Nucb2* mRNAs in mice pituitary (Fig. 7E,F).

### Disruption of Nucb1 caused minimal effects to the expression of steroidogenic enzyme mRNAs in the adrenal gland and Pomc, Pcsk2 and Crhr1 in pituitary

The abundance of steroidogenic enzyme mRNAs in the adrenal gland, and HPA-related mRNAs in the pituitary gland of *Nucb1* KO mice are shown in Tables 1, 2. The genetic disruption in *Nucb1* did not affect the steroidogenic enzyme mRNA abundance in male and female mice, except for the downregulation of *Hsd3β* in female *Nucb1* KO mice (Table 1). Moreover, the expression of *Pcsk1* in adrenal gland were significantly lower in male *Nucb1* KO (Table 1). The expression of *Pomc*, *Pcsk2* and *Crhr1* in the pituitary of *Nucb1* KO mice of both sexes (Table 2) was unaffected. *Nucb2* was upregulated in the adrenal gland samples of *Nucb1* KO mice of both sexes (Table 1). *Mc2r* mRNA in the adrenal gland (Table 1) and *Nucb2* mRNA in the pituitary were unaffected by the disruption of *Nucb1* in mice of both sexes (Table 2).

**Table 1:** A comparison of mRNAs in the adrenal gland of wildtype (WT) vs *Nucb1* KO mice. Studies were conducted on 6 tissue samples/mice per group. No change, an increase, or a decrease in the knockout male or female mice when compared to their respective control groups is denoted in the tables.

*mRNA*	WT versus *Nucb1* KO	
	Male	Female
*Star*	No change	No change
*Cyp21*	No change	No change
*Cyp11a1*	No change	No change
*Cyp11b1*	No change	No change
*Cyp11b2*	No change	No change
*Hsd3β*	No change	Decrease
*Mc2r*	No change	No change
*Nucb2*	Increase	Increase
*Pcsk1*	Decrease	No change

**Table 2:** HPA axis-related and endogenous Nucb2 mRNAs in the pituitary of wildtype (WT) and *Nucb1* KO mice (n = 6 samples or mice/group). No change, an increase, or a decrease in the knockout male or female mice when compared to their respective control groups is denoted in the tables.

*mRNA*	WT versus *Nucb1* KO	
	Male	Female
*Pcsk2*	No change	No change
*Pomc*	No change	No change
*Crhr1*	No change	No change
*Nucb2*	No change	No change

## Discussion

This research aimed to determine whether NUCB1/NLP and NUCB2/NESF-1 exist in a human adrenal cortex cell line, and studied if they act directly on steroidogenic enzymes to regulate cortisol synthesis. NUCB2/NESF-1-like immunoreactivity was detected in the cytoplasm and nucleus of human adrenal cortex cells. NUCB2/NESF-1 immunoreactivity was found in the cytoplasm murine neurons^1,21^ and gastric cells^22^ and rat brain^23^ and Leydig cells^24^, and the human brain^25^. In another study, NUCB2/NESF immunoreactivity was observed H295R cell cytoplasm^26^. This agrees with our results showing cytoplasmic staining of NUCB2/NESF in the same cells. We also NUCB2/NESF immunoreactivity in the nucleus of H295R cells. Previously, NUCB2 immunoreactivity was detected in the nucleus of endothelial cells of mouse lung^27^. Given that NUCBs are DNA binding proteins^1^, the staining found in the nucleus is reflective of that role. NUCB1/NLP-like immunoreactivity was reported in the central nervous system and peripheral tissues, including endocrine cells of the anterior pituitary of rats and mice^1,28^, mouse insulinoma MIN6 cell line and mouse pancreatic beta cells^17^ as well as testis, ovary, and head kidney interrenal cells^2^ and pituitary of goldfish^18^. The results reported here show that human adrenal cortex cells express NUCB peptides and possibly other bioactive peptides residing within it, which may act directly on these cells to regulate the steroidogenesis pathway and cortisol synthesis. This suggests that the adrenal gland is likely another tissue source of circulating NUCB and peptides processed from it. The role of locally produced NUCB peptides warrants further consideration.

Immediately after the discovery of NESF-1^29^ and its role in metabolic regulation, several studies have examined its implication in anxiety and stress hormone synthesis in vitro and in vivo^30^. NESF-1 stimulates ACTH secretion from goldfish dispersed pituitary cells in vitro^2^. NESF-1 and NLP also stimulate the synthesis of CRH and ACTH in the hypothalamus and anterior pituitary^1,2,12^. However, in the current study, the synthesis and release of cortisol was adversely influenced by NESF-1 and NLP. Cortisol is a catabolic hormone that stimulates oxidation of lipids and decreases the synthesis of lipids and carbohydrates to create a surge in available energy. CRH-stimulated cortisol in humans stimulates the consumption of food^8^. Next, we studied whether NESF-1 and NLP affect steroidogenic enzymes involved in human cortisol synthesis in the adrenal gland. In agreement with our in vitro synthesis and secretory studies described above, both NLP and NESF-1 decreased the expression of most steroidogenic enzymes involved in cortisol synthesis at 12 h post-incubation. Meanwhile, ACTH, as a positive control, increased the expression of these enzymes. These results were complemented by the decreased cellular cortisol content observed in NLP and NESF-1 treated cells and increased cortisol content found in ACTH treated cells at 24 h post-incubation. Another study showed that NESF-1 inhibits the growth of and triggers the apoptosis of H295R cells through *BAX*, *BCL-XL* and *BCL-2* genes as well as extracellular signal-regulated kinase (ERK1/2), p38 and Jun N-terminal protein kinase (JNK1/2) signalling cascades. These effects of NESF-1 possibly contributed to the suppression of cortisol secretion^26^. We did not see any cell death in our in vitro studies. Since the doses tested and durations of incubation in this research and the above discussed study are different, a direct comparison of results from these two studies are not possible.

In goldfish, ICV and IP administration of NESF-1 increased cortisol secretion and *crh* mRNA level in vivo and ACTH secretion in vitro^2^. ICV administration of NESF-1 enhanced the secretion of ACTH and corticosterone in rats^12^. In these studies, the net stimulatory effect of NESF-1 on cortisol release seems to be through the ACTH-dependant pathway. The results of this study, although solely using a human cell line, show that NLP and NESF-1 could act directly on the adrenal cortex to inhibit cortisol synthesis and secretion. This effect is ACTH independent. However, as reported in previous studies, both NESF-1 and NLP stimulated POMC synthesis in mouse corticotrophs^1^ and NESF-1 stimulated ACTH secretion in goldfish pituitary cells^2^. Therefore, this positive effect of NES-1 and NLP on ACTH synthesis and secretion likely negates any adverse effect these peptides have on cortisol synthesis and secretion in vivo. This is possibly a reason why cortisol was found elevated in vivo after NESF-1 treatment. This comparison has limitations as route of administration, peptides used, doses and duration of treatment tested all influence the outcomes of these studies. The current study also showed that *MC2R* mRNA expression was increased by ACTH at 12 and 24 h post-incubation in H295R cells (Supplementary Information). The low response to ACTH has been a drawback of H295R cells, which was attributed to low levels of MC2R in these cells^31^. In agreement with current findings, other studies showed that H295R cells showed higher basal *MC2R* expression levels and greater response to ACTH stimulation at time points above 12 h^32^. Another interesting piece of evidence from these results is the inhibitory effect of NLP and NESF-1 on endogenous *NUCB* mRNA expression, while ACTH stimulated *NUCB1* and *NUCB2* in human adrenal cortex cells. This effect of NLP and NESF-1 are likely due to a negative feedback on endogenous NUCBs due to the excess levels of processed peptides sensed by the cells during the in vitro treatment with synthetic peptides.

NLP and NESF-1 act through PKA/CREB mediated pathway to stimulate POMC synthesis in mouse corticotrophs^1^. Similarly, ACTH stimulates the production of GCs through MC2R-cAMP/PKA/CREB pathway in the adrenal cortex cell line, including Y1 mouse adrenocortical cells^33^. We hypothesized that NLP and NESF-1 modulate cortisol synthesis through the CREB-mediated pathway in human adrenal cortex cells. To assess the potential signalling pathway that mediates the inhibitory effect of NLP and NESF-1 on cortisol synthesis, the phosphorylation of CREB at different time points was first checked. In the current study, these peptides decreased P-CREB/T-CREB ratio at 90 min post-incubation. In the next step, we found that when H295R cells were preincubated with NLP and NESF-1, the PKA/CREB signalling-mediated cortisol secretion by forskolin was significantly decreased. These results demonstrate that AC/PKA/CREB pathway is critical to mediate the effect of NUCB-derived peptides on cortisol synthesis in human adrenal cortex cells. One report showed that NESF-1 stimulated intracellular calcium influx in hypothalamic neurons of rats, and this effect was abolished by pertussis toxin, which suggests that NESF-1 interacts with G-protein coupled receptor [GPCR] in brain cells^34^. CF568-labeled-NESF-1 and CF568-labeled-NLP bind to the membrane of GH3 cells, suggesting a possible GPCR-mediated action of NESF-1 and NLP in these cells^28^. A putative nesfatin-1 receptor GPCR12-like immunoreactivity was also detected in the pituitary and interrenal cells of goldfish^2^. As a result, these results and previous findings suggest the presence of a functional receptor on the surface of stress-related cells for NESF-1 and NLP.

We also studied the effect of IP injection of NLP on the genes as mentioned above in adrenal gland and pituitary of WT mice. There have been several studies on the administration of NESF-1 in animal models and its effect on either stress-related hormone synthesis and gene expression patterns or the emergence of anxiety-like behaviour. Repeated IP injection of NESF-1 induced anxiety-like behaviour in rats^35^. The increased anxiety-like behaviour was associated with elevated NUCB2/NESF-1 in the hippocampus, plasma and stomach of rats following the sequential stress induction^36^. IP and ICV administration of NESF-1 increased cortisol release and hypothalamic *crh* mRNA levels in goldfish, respectively^2^. However, in the current study, IP injection of NLP decreased the expression of steroidogenic enzymes [in agreement with the in vitro results], while increasing the expression of *Pomc* and *Pcsk1* mRNA levels in mice pituitary. The level of endogenous *Nucb1* and *Nucb2* were decreased in the adrenal gland, and pituitary samples of NLP injected mice. These results show that NLP and NESF-1 regulate cortisol synthesis and steroidogenesis through ACTH- dependant and independent manner, respectively.

The genetic disruption of *Nucb1* did not affect the genes mentioned above except for the downregulation of *Hsd3β* in the adrenal gland of female *Nucb1* KO and *Pcsk1* in the pituitary of male *Nucb1* KO mice. These results were linked with tissue-specific upregulation of *Nucb2* in the adrenal gland, while no changes were observed in the pituitary of male and female *Nucb1* KO mice. The results of this study demonstrate that the expression of most steroidogenic enzymes remained unaffected in *Nucb1* disrupted mice. This is possibly due to the compensatory upregulation of *Nucb2* in the adrenal gland, which was detected in current research. The stress -related gene expression pattern in *Nucb1* KO mice appears to be sex- and tissue-specific. Previous studies emphasized the predominant implication of NUCB2/NESF-1 in the mediation of anxiety in a sex-dependent manner. One study showed that circulating NESF-1 level in female patients was significantly correlated with the increased severity of stress, while no correlation was observed in male patients^37^. Another study depicted the sex-dependant regulation of NUCB2/NESF-1 under depressive conditions with the positive correlation of circulating NESF-1 with an increased degree of depression in obese female patients. In contrast, such correlation was not observed in male patients^38^. Since the H295R cells is originated from a female patient, the pattern of mRNAs encoding steroidogenic enzymes and endogenous *Nucbs* found in this study can be interpreted as a female-specific effect. Since the endogenous hormonal milieu is absent, this in vitro approach has limitations. Male cells and in vivo studies using both males and females are crucial to confirm the conclusions reached here.

In summary, this study provides the first evidence on the direct and indirect effects of NLP and NESF-1 on cortisol synthesis and steroidogenesis, and how genetic disruption of *Nucb1* versus the injection of NLP affects HPA-related gene expression. The results of current study show that NLP and NESF-1 directly inhibit GC synthesis in an ACTH-independent manner. This report, along with previous publications expand the current understanding of NLP and NESF-1 as stress-responsive peptides and their role in the regulation of stress hormones synthesis. More studies on stress in response to NESF-1/NLP administration in different species are warranted. The role of NLP on the stress/HPA axis in humans is poorly understood. The implications of the results reported here on human diseases including anxiety and depression warrant further consideration. The outcome of this study and other studies set the stage to further explore NUCB peptides present within them in stress and anxiety in animals. They also raise possibilities for future studies aimed at its use in animals and humans as a biomarker or as a target for stress-related disorders.

## Methods

### Cell culture

Human adrenal cortex cell line [H295R, CRL-2128™] was purchased from ATCC. This cell line was derived from the NCI-H295 pluripotent adrenocortical carcinoma cells [ATCC CRL-10296]. The cells were grown as per the protocols provided by the supplier in 10 cm cell culture dish containing DMEM:F12 Medium [cat no. 30–2006, ATCC] supplemented with 1% ITS + Premix [cat no. 354352, Corning, Canada] and 2.5% Nu-serum [cat no. 355100, Corning, Canada], at 37 °C in 5% CO2 and 95% humidified atmosphere. When cells reached confluency, they were split at the desired density and used for specific experiments.

### Peptides

Rat NES-1 (Cat no# 003-22B) was purchased from (Phoenix Pharmaceuticals INC. CA, USA). Murine NLP (> 95% purity) was custom synthesized (ABGENT, USA). The sequence of NESF-1 is: H-vpidvdktkvhnvepvesarieppdtglyydeylkqvievletdphfreklqkadieeirsgrlsqeldlvshkvrtrldel and NLP is: H-VPVDRAAPPQEDSQATETPDTGLY YHRYLQEVINVLETDGHFREKLQAANAEDIKSGKLSQELDFVSHNVRT KLDEL. The putative bioactive core of NESF-1 and NLP are very highly conserved across human and rodent peptides, and our preliminary results found equipotent activity for these peptides in human cells.

### Immunocytochemistry

Localization of NUCB1/NLP and NUCB2/NESF-1 in H295R cells was carried out using immunocytochemistry [ICC] based on previously reported protocols^1^. The primary antibodies used in this study were rabbit anti-mouse NUCB1 [1:200, custom synthesized, cat no. 1312-PAC-02, Pacific Immunology, USA], rabbit anti-mouse NUCB2 [1:200, RRID: AB_2891124, cat no. 1312-PAC-01, Pacific Immunology, USA]. Secondary antibodies in this study were goat anti-rabbit Alexa Fluor 488 [1:200, RRID: AB_2630356, cat no. ab150077, ABCAM, UK]. No primary antibody was considered as a negative control group. The imaging was conducted using an Olympus DP70 camera and an Olympus BX51 microscope. Immunostaining obtained is referred to as NUCB1/NLP and NUCB2/NESF-1 to represent precursors and the encoded peptides detected by the antibodies used.

### Cortisol measurement

Cortisol was measured in cell lysate and cell culture media. For this purpose, H295R cells were seeded in 6 well plates and treated with NLP/NESF-1/ACTH [10 nM for peptides and 100 nM for the positive control group] for different time points, including 6 h, 12 h and 24 h. Then, cells were washed with cold PBS and trypsinized. After centrifugation at 15,000 *g* at 4 °C, trypsin–EDTA was removed, and cells were suspended in cold PBS and sonicated 3 times for 1–2 min. Cell culture media from all groups were collected to measure the secreted cortisol level in the media using human cortisol ELISA kit [cat no. COR31-K01, Eagle Bioscience, Inc, USA] according to manufacturer’s instructions. All cell lysate samples were diluted with cold PBS. The assay sensitivity and dynamic range test were 0.4 μg/dL and 0.5–60 μg/dL, respectively.

### Mechanism of action of NLP and NESF-1 on H295R cells

We reported that NLP and NESF-1 act through AC/PKA/CREB pathway to regulate POMC synthesis in mouse corticotrophs^1^. ACTH stimulates the production of GCs through MC2R-cAMP/PKA/CREB pathway in the adrenal cortex cell line, including Y1 mouse adrenocortical cells^32^. Therefore, we hypothesized that NLP and NESF-1 modulate cortisol synthesis through the CREB-mediated pathway in human adrenal cortex cells. To determine the cells signalling mediators, H295R cells were incubated with fresh media [control group] or NLP/NESF-1 [experimental groups] at effective doses [10 nM for peptides and 100 nM for ACTH] for 90 min, and then phosphorylated [P]-CREB/total [T]-CREB ratio was assessed in the cell lysate.

In the second part of this study, H295R cells were preincubated with NLP or NESF-1 to block the CREB-mediated pathway for 12 h, then forskolin [cat no. 11018–5, Cayman Chemical Co, USA] as a specific adenylate cyclase stimulator [10 µM] was added to the media for 12 h. The cortisol level in cell lysate or cell culture media was measured using a cortisol ELISA kit as explained below. The dissolved forskolin in DMSO was less than 0.1% in cell culture media. The concentration and time point for forskolin incubation were chosen based on the recommended dose range in the supplier catalogue and were independently validated in pilot studies [data not shown]. ACTH treated cells were used as a positive control group.

### Effects of NLP and NESF-1 on steroidogenic enzymes and endogenous Nucb mRNAs in H295R cells

H295R cells at a confluency of 90% were treated with mouse NLP or rat NESF-1 at different concentrations [1–10 nM] for different time points [6 h and 12 h]. Human ACTH [cat no. 001–01, Phoenix Pharmaceuticals Inc., USA] at 100 nM was used as a positive control group. After the incubation period, cells were harvested, and the abundance of mRNAs for steroidogenic enzymes was assessed using qPCR. The abundance of endogenous *Nucb* mRNAs was also measured. The effective dose and time points were selected based on pilot studies [data not shown] and previous research^1^. In our previous study, we assessed the identity and similarity percentage between rat and mice peptides. Interestingly, rat and mouse NES-1 have 97.6% identity and 98.8% similarity in amino acid sequence. In addition, rat and mouse NLP share 97.4% identity and 98.7% similarity in amino acid sequence^1^.

### Intraperitoneal NLP injection in wildtype mice and Nucb1 knockout [KO] mice

For the first study, C57BL/6J wildtype [WT] mice [n = 6 mice/group; both males and females; based on power analysis and reduced number of animals based on animal care approval] were used to study whether intraperitoneal [IP] injection of NLP affects HPA-related hormones and genes. All mice were fasted for 4 h [from 9 am] before the commencement of experiment. Mouse NLP was dissolved in sterile saline [0.9% NaCl] and then diluted to 100 µg/kg BW. It was administered to mice using insulin syringes [BD®, cat no. 324911] attached to a 27G needle in the lower right quadrant of abdomen to avoid damage to internal organs. The selected dose was validated to be metabolically active and decrease food intake and modulate body weight [14]. After 30 min, mice were euthanized, and blood samples and internal organs were collected.

To characterize the effects of disruption of *Nucb1* in the second study, breeding pairs of homozygous C57BL/6NCrl-Nucb1em1[IMPC]Mbp/Mmucd mice (global *Nucb1* disrupted mice) were purchased from the University of California [generated by Dr. Kent Lloyd, Mouse Biology Program, University of California-Davis, USA]. These breeding pairs were used to establish a colony of homozygous *Nucb1* disrupted mice. In these mice, the exon 4 and flanking splicing regions of *Nucb1* were constitutively deleted using CRISPR Cas9 gene editing technology in C57BL/6J mouse zygotes. All the animals were housed under 12 h light:12 h dark cycle [7 am–7 pm], humidity [30–60%], and temperature [18–22 °C] controlled vivarium located in the College of Medicine Lab Animal Services Unit, University of Saskatchewan. All protocols were prepared based on guidelines of the Canadian Council for Animal Care and were approved by the University of Saskatchewan Animal Care Committee [2012–0033]. The experiments conducted adhered to the ARRIVE guidelines on animal use and care. Age-matched mice were chosen and fed a standard rodent chow diet [cat no. 500I, LabDiet, 7.94% carbohydrate, 28.67% protein, 13.38% fat, Energy density = 4.09 kcal/g]. Mice were anesthetized using 3% isoflurane inhalation and were euthanized by cervical dislocation. Different tissues, including the adrenal gland and pituitary, from these mice were collected after cervical dislocation, total RNA was extracted, and the expression of stress-related genes was assessed in the cell lysate.

### Total RNA extraction, cDNA synthesis, and real-time quantitative PCR

This section was carried out as described in previously reported protocols [1]. Briefly, RNA was extracted using Ribozol [cat no. N580, VWR, USA], and then RNA’s quantity and quality were determined by NanoDrop 2000 [Thermo Fisher Scientific]. The RNA was reverse transcribed to cDNA using iScript Reverse Transcription Supermix for RT-qPCR [cat no. 170884, Bio-Rad, USA] followed by the quantitative measurement of mRNA expression by qPCR in a CFX Connect Optic module [Bio-Rad, USA] following the requirements of the MIQE guidelines [17] and using SensiFAST™ SYBR No-ROX MIX [cat no. BIO-98050, Bioline, UK]. The primers sequences and annealing temperatures are listed in Table S1 (Supplementary Information) purchased from Integrated DNA Technologies [IDT]. Three negative controls, including no template DNA [NTC control], no reverse transcriptase control from cDNA synthesis process [RTC control], and a nuclease-free water sample [PCR control], were also included for each gene expression study. Thermal cycling set-up for all genes was the following: denaturation [95 °C for 5 s], annealing [specific for each primer for 25 s] and elongation [60 °C for 25 s], 35 cycles. At least 3 independent experiments with triplicates for in vitro studies [final samples ≥ 9] and more than 6 mice/group/sex were considered. The abundance of mRNA was calculated based on the Pfaffl method and gene-specific efficiencies [18], relative to the geometric means of the 2 housekeeping genes.

### Western blot analysis

The western blot protocol followed was described previously^1^. Briefly, total protein was extracted using RIPA Lysis buffer [cat no. 89901, Thermo Fisher Scientific, USA] and the protein concentration was determined by Bradford assay. An equal amount of crude protein [40 μg] was electrophoresed on 8–16% Mini-Protean TGX gels [cat no. 456–1104, Bio-Rad, USA], and the protein bands were transferred to nitrocellulose membranes [cat no. 1704158, Bio-Rad, USA] using the Trans-Blot Turbo Transfer System [Bio-Rad, USA]. After blocking the membranes, they were incubated with primary antibodies overnight at 4 °C followed by washing steps, incubation with secondary antibodies for 1 h and final washing. Then the membranes were visualized by ChemiDoc MP Imaging System [Bio-Rad, USA]. The band intensity was analyzed by ImageJ [National Institutes of Health, Bethesda, MD, USA].

Primary antibodies used in this study were monoclonal mouse anti-beta-actin antibody [1:1000, RRID: AB_528068, cat no. JLA20, Developmental Studies Hybridoma Bank, DSHB, University of Iowa, USA], polyclonal rabbit anti-phospho [Ser 133]-CREB antibody [1:1000, RRID: AB_2561044, cat no. 9198, Cell Signalling, USA], monoclonal rabbit anti-CREB antibody [1:1000, RRID: AB_331277, cat no. 9197, Cell Signalling, USA]. The secondary antibodies used were goat anti-rabbit IgG [H+ L]-HRP conjugate antibody [1:5000, RRID: AB_11125142, cat no. 170–6515, Bio-Rad, USA] and goat anti-mouse IgG [H + L]-HRP conjugate antibody [1:5000, RRID: AB_11125547, cat no. 170–6516, Bio-Rad, USA].

### Statistical analysis

Statistical analysis was conducted using the SPSS statistical software [IBM SPSS Statistics for Windows, Version 23.0, USA]. The normality of data distribution was analyzed by Shapiro–Wilk’s test and all data were normally distributed. The homogeneity of variances was checked by Levene’s test. The significance level was set at p < 0.05. The single comparison was performed by Student’s *t*-test, and multiple comparisons were performed by one-way ANOVA followed by Tukey’s multiple comparison test. All graphs were plotted by GraphPad Prism [GraphPad Software, Inc., Prism 8 for Windows, Version 8.4.2, USA].

### Supplementary Information

Supplementary Information.

## Data Availability

The datasets generated during and/or analysed during the current study are available from the corresponding author on reasonable request.
