# Supplementary material for: Suppressive action of nesfatin-1 and nesfatin-1-like peptide on cortisol synthesis in human adrenal cortex cells
Source: Sci Rep. 2024 Feb 17;14:3985. doi: 10.1038/s41598-024-54758-7 (PMC10874440; doi:10.1038/s41598-024-54758-7)
Supplement: Supplementary file 1 — Supplementary Information. [file 41598_2024_54758_MOESM1_ESM.docx]

**Suppressive action of nesfatin-1 and nesfatin-1-like peptide on cortisol synthesis in human adrenal cortex cells**

**Authors:**

Atefeh Nasri^1^, Jade Sands^1^, Suraj Unniappan^1*^

**Institutional affiliations:**

^1^Laboratory of Integrative Neuroendocrinology, Department of Veterinary Biomedical Sciences, Western College of Veterinary Medicine, University of Saskatchewan, Saskatoon, Saskatchewan S7V 1H2, Canada

*Corresponding author

**Supplementary Material**

**Table S1.** The list of primer forward and reverse sequences and their annealing temperature

**Figure S1.** The expression of MC2R in H295R cells incubated with ACTH at different time points

**Figure S2.** Full size western blot gel images shown in Figure 3

**Table S1.** The list of primer forward and reverse sequences and their annealing temperature

| Gene | Accession no. | Primer sequence (5′−3′) | | Tem (°C) |
| --- | --- | --- | --- | --- |
|  |  | Forward | Reverse |  |
| HM-*STAR* | NM_000349.3 | CTCTCTACTCGGTTCTCGGC | AGCCCTCTTGGTTGCTAAGG | 59.3 |
| HM-*CYP11A1* | KU178011.1 | GGTGACAATGGCTGGCTAAAC | GTTGCCGAGCTTCTCCCTGTAA | 60.5 |
| HM-*CYP17A1* | NM_000102.4 | TGGCTCTCTTGCTGCTTACC | ACGAACCGAATAGATGGGGC | 60 |
| HM-*CYP21* | XM_047443023.1 | GCAGACCTGAGCCACTTACC | CACAGAACTCCTGGGTCAGC | 60 |
| HM-*CYP11B1* | NM_000497.4 | AGGAGACCTTGCGGCTCTAC | GAGTAGAGGAACACGCGCA | 60.6 |
| HM-*CYP11B2* | AH001448.2 | TTGTTCAAGCAGCGAGTGTTG | GCATCCTCGGGACCTTCTC | 59.5 |
| HM-*HSD3β* | NM_001166120.2 | CCACACCGCCTGTATCATTG | CCCGGCTACCTCTATGCTAC | 59.6 |
| HM-*ACTβ* | HQ154074.1 | TCCACGAAACTACATTCAATTCCA | ACAGAGTACTTGCGCTCAGGA | 60 |
| HM-*GAPDH* | NM_001357943.2 | TGCACCACCAACTGCTTAGC | GGCATGGACTGTGGTCATGAG | 61 |
| HM-*NUCB1* | XM_017026845.2 | GACCCTCAGAACCAGCATACA | CTCCCAGTGACTCCAGATAACG | 60 |
| HM-*NUCB2* | XM_047426994.1 | AGTAGATGAGGTGGAGGACCA | TAGGCACAGCTTCAAGAGCA | 59 |
| MS-*Star* | L36062.2 | TCACTTGGCTGCTCAGTATTGAC | GCGATAGGACCTGGTTGATGA | 60 |
| MS-*Cyp21* | NM_009995.2 | GGTTCCAGGAAGCGATCTG | CAACTAGGGCTAGCAGCATC | 57 |
| MS-*Cyp11a1* | NM_001346787.1 | GACCTGGAAGGACCATGCA | TGGGTGTACTCATCAGCTTTATTGA | 59.3 |
| MS-*Cyp11b1* | NM_001033229.3 | CGCTGCAAATCCTCAGAAGG | ACATTGAGGACTGTCCCAGCA | 60.1 |
| MS-*Cyp11b2* | NM_009991.4 | TGGCATTGTGGCGGAACTAA | AAGGGGATTGCTGTCGTGTC | 60.4 |
| MS-*Hsd3β* | XM_006501036.3 | CTTTTCAGCCACCACCATCT | GGTCTGTCCTTCCCAGTGAT | 58.5 |
| MS-*Mc2r* | NM_001301372.1 | CACACCAATGACACCGCAAG | CACAGCCAGGAGGACAATCA | 60 |
| MS- *Pcsk1/3* | NM_013628 | AGGTCGAGTCTAGCTGGTGT | TGCTCCATGGCTCAAAACCT | 60 |
| MS-*Pcsk2* | NM_008792 | CAGCTGGCGTGTTTGCATTA | AATTCCAGGCCAACCCCATT | 60 |
| MS-*Crhr1* | AB375515.1 | CCTCCAGGATCAGCAGTGTG | GTGTTGTAGCGGACACCGTA | 60 |
| MS-*Nucb1* | XM_006540696.4 | CACGGGCCTGTACTACCAC | TTGGCTCAGCTTTCCACTCT | 60 |
| MS-*Nucb2* | XR_004934144.1 | AACACGAGCGGAGAGAGTAT | AGGGTCCAATCCATCAGTCT | 60 |
| MS-*Actβ* | BC138614 | CCACTGCCGCATCCTCCTCC | CTCGTTGCCAATAGTGATGAC | 60 |
| MS-*Gapdh* | XM_036165840 | GACATCAAGAAGGTGGTG | ATACCAGGAAATGAGCTTGACAAA | 59 |


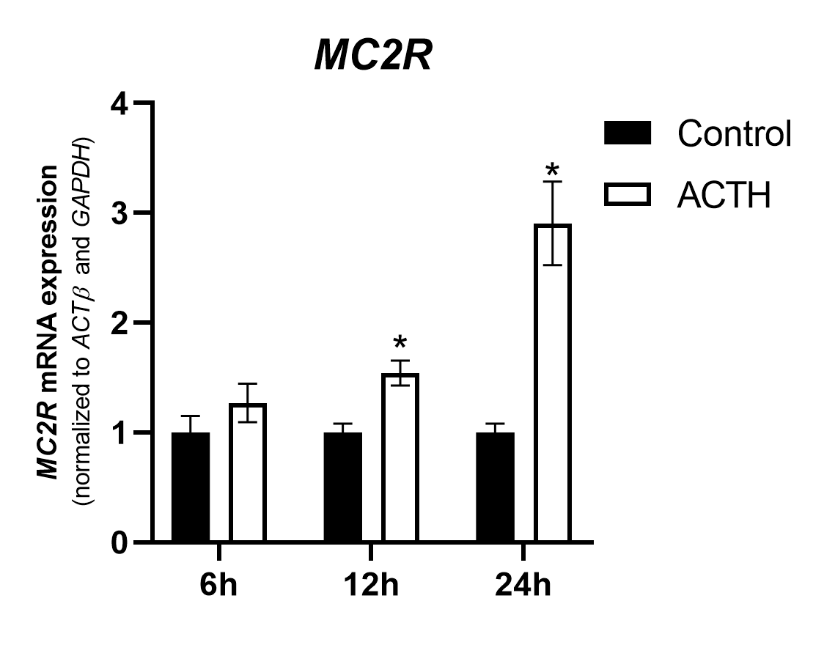


**Figure S1.** The expression of MC2R in H295R cells incubated with ACTH at 6, 12 and 24 h. The results presented are pooled from 3 independent studies with at least triplicates for each treatment. Asterisks show statistical differences between experimental and control groups.


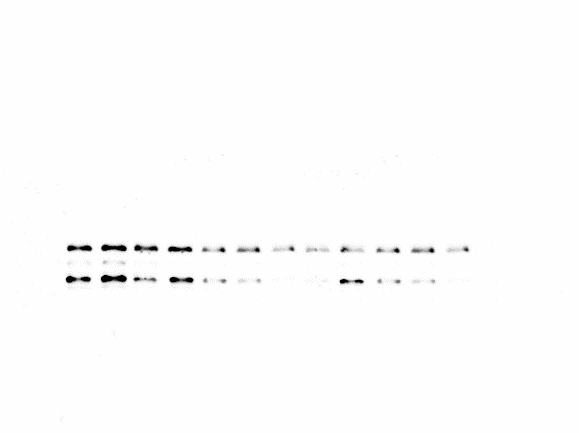

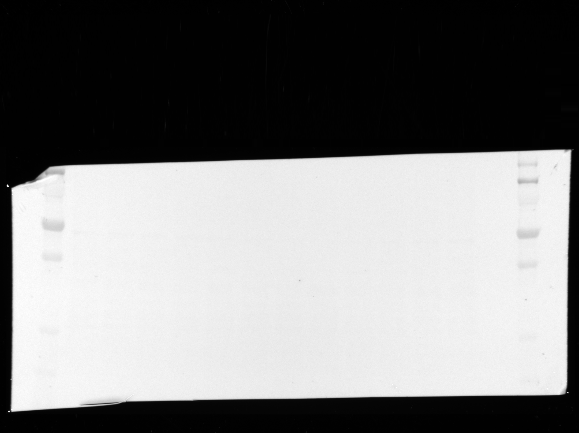

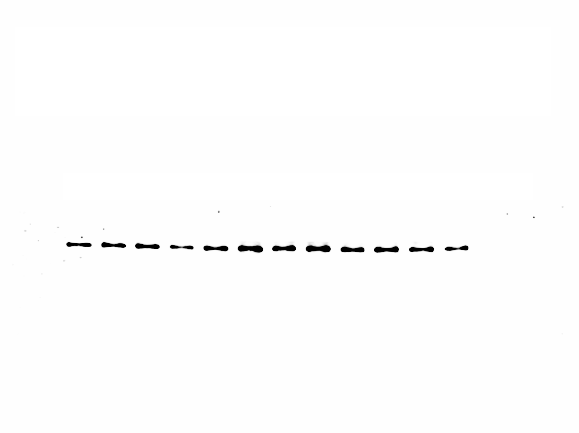

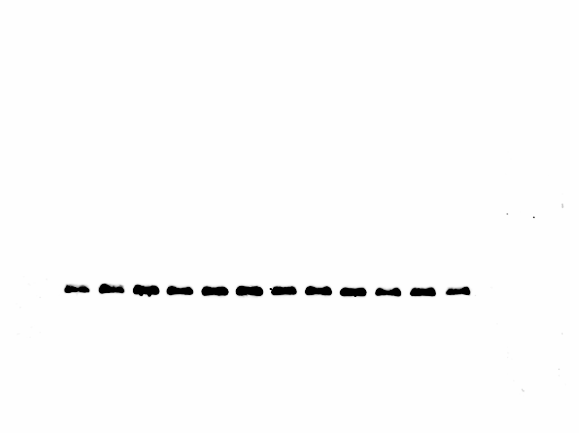


**H295R cells**

**C NLP NESF-1**

**C NLP NESF-1**

**C NLP NESF-1**

37KD

50KD

**T-CREB**

**43kD**

**P-CREB**

**43kD**

**Beta-ACTIN**

**45kD**

**Figure S2.** Full size Western blot gel images shown in Figure 3 of the manuscript. The areas within the dark rectangles were cropped and are shown in Figure 3. The protein ladder used to size the bands are shown in the upper right image.

**Images of same Figure S2 blots shown earlier with higher exposure. Background and margins are visible in these images.**


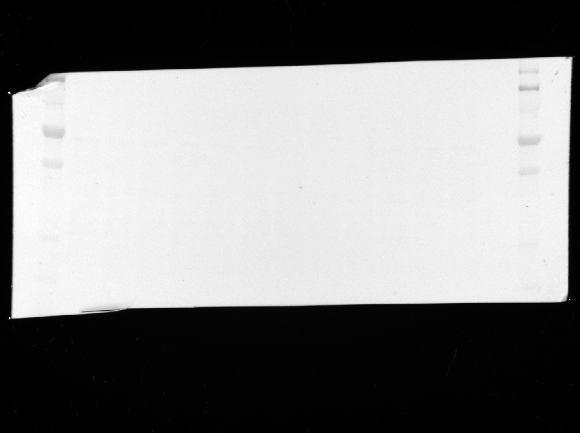

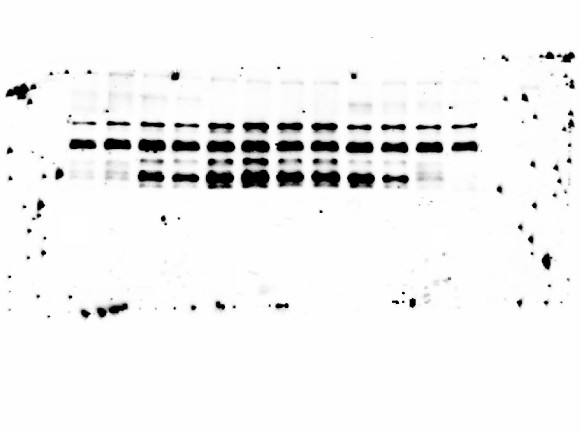


**Beta-ACTIN**

**45kD**

50KD

**C NLP NESF-1**


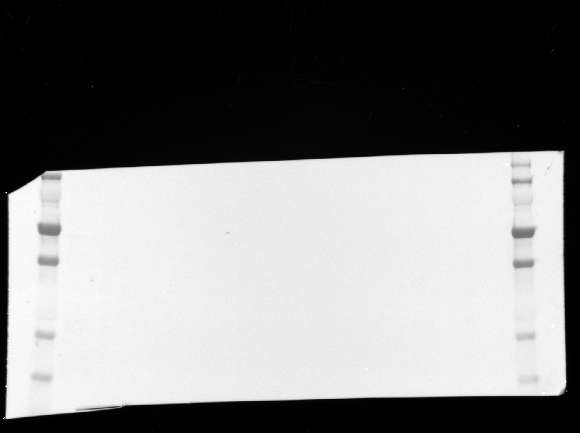

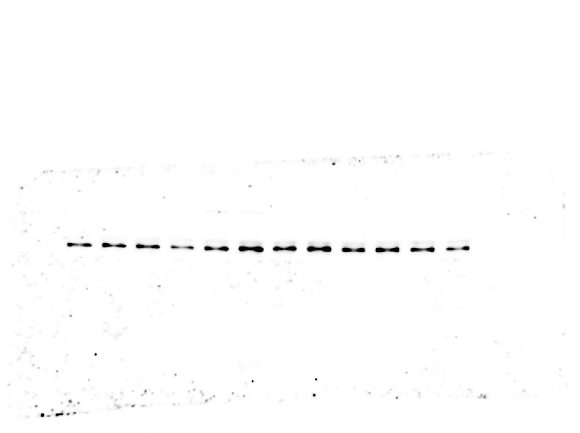


**C NLP NESF-1**

50KD

**T-CREB**

**43kD**


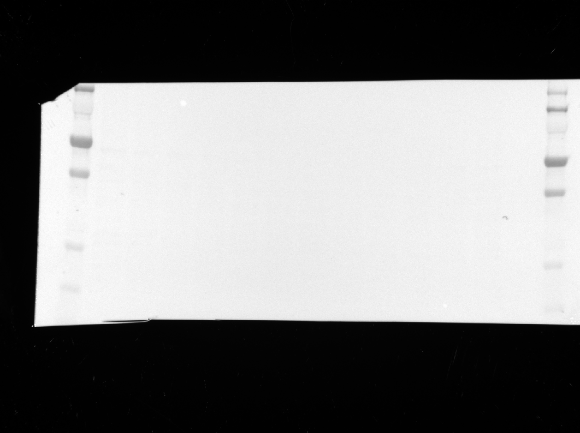

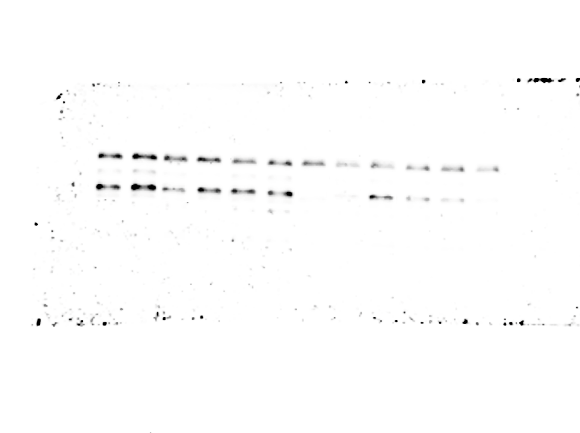


50KD

**P-CREB**

**43kD**

**C NLP NESF-1**
